# Ontology-based annotation and fuzzy recommendation for community formation in smart city knowledge platforms

**DOI:** 10.3389/frai.2026.1832804

**Published:** 2026-06-10

**Authors:** Amjad Hawash, Paolo Bottoni, Danilo Avola

**Affiliations:** 1 Department of Information and Computer Science, An-Najah National University, Faculty of Information Technology & Artificial Intelligence, Nablus, Palestine; 2 Department of Computer Science, Sapienza - University of Rome, Rome, Italy

**Keywords:** community detection, fuzzy logic, matching, recommendation, relevance tuning, smart cities, web annotation

## Abstract

**Introduction:**

The MADCOW annotation system enables group-based annotation, allowing users to direct annotations toward communities focused on specific domain topics. In smart city environments, such groups may include citizens, urban planners, and domain experts collaborating on urban services, infrastructure, mobility, environment, and public safety. Existing recommendation approaches mainly rely on ontology-based semantic similarity, which limits their effectiveness in dynamic collaborative settings.

**Methods:**

To address this limitation, this study extends ontology-based matching by incorporating behavioral and structural factors, where user activity is modeled using the number of posted annotations and joined groups, and group relevance is represented by group size and membership growth rate. These heterogeneous features are integrated with ontology-based semantic similarity using fuzzy logic operators to construct a more flexible and expressive ranking framework.

**Results:**

Experimental evaluations demonstrate that the proposed approach improves the quality of user-to-group and group-to-user ranking compared to methods relying solely on ontology-based graph similarity measures. However, the model's performance may decrease in scenarios with highly sparse annotation data, indicating the need for further investigation into robustness under data-scarce conditions.

**Discussion:**

The aggregation of annotations and of users according to topics of interests may facilitate focused discussions about issues related to city governance among concerned citizens. The identification of active groups and users may lead to more effective recommendations for user enrolment.

## 1 Introduction

The technologies at the basis of the so-called Web 2.0 and the possibilities they offer for capturing users' interests have led to the diffusion of recommender systems and their integration with almost all websites, with significant support for the production of

user-generated content (from e-commerce portals to social networks), suggesting all kinds of experiences (from holidays abroad to recommended reading).

Similar technologies have also played a major role in the development of smart city platforms in recent years (Yang et al., 2025). These platforms enable citizens, urban planners, and domain experts to collaboratively exchange knowledge, discuss urban challenges, and contribute to improving city services such as public safety, transportation, and environmental monitoring. In this context, annotation mechanisms represent a powerful means of capturing user-generated knowledge, as they allow individuals to comment on digital resources related to urban infrastructure, regulations, environmental data, and community initiatives (Leclercq and Rijshouwer, 2022). Such annotations provide a rich basis for identifying shared interests among users and facilitating collaborative knowledge exchange. Furthermore, groups within annotation-based systems can represent communities centered around specific urban themes, such as sustainable mobility, energy efficiency, urban planning, or environmental sustainability (Kozhevnikov and Svitek, 2025). By leveraging semantic descriptions of both urban topics and user-generated annotations, it becomes possible to automatically identify common interests, recommend relevant users and groups, and foster the emergence of collaborative communities within smart city ecosystems (He et al., 2025).

Annotations of Web pages can also support practical recommendation scenarios. For example, users might be interested in being aware of the presence of annotations on pages from a website for which they have also annotated some pages, or on pages that are somehow related to topics on which they have already produced annotations. Similarly, finding that another user has produced annotations characterized by the same sentiment as ours might bring us to explore the set of annotations produced by that user.

Despite the widespread usage of annotation-based recommender systems in web platforms and smart city environments, it is still challenging to create cohesive groups and identify users who are interested in a certain group. Current approaches that rely on semantic matching between user-generated tags and domain ontologies make the assumption that similarities in annotated content are sufficient to produce meaningful recommendations.

However, we experimentally observed that this assumption may result in a decrease of the quality of recommendations when user behavior and group dynamics are disregarded. Indeed. the utility of a recommendation in real-world collaborative contexts can be influenced by variables such as user activity, engagement level, and group evolution.

These considerations led to extending the **MADCOW**^1^ interactive system for annotation of Web pages (Bottoni et al., 2004) with the possibility of forming groups of users (Avola et al., 2010, 2017), thus allowing them to make their annotations public only within a group whose members share some common interest with which the annotation is concerned. As a result, it becomes possible to entertain focused discussions on the annotated pages.

Based on this context, in this paper we address two fundamental problems: (1) how to effectively identify relevant users for a given group; (2) how to recommend appropriate groups to a given user.

To tackle these issues, explorations were conducted to identify measures that could assess the relevance of the annotations produced by a user concerning the kind of annotations appearing (or expected to appear) in a group; see, e.g., Avola et al. (2014a,b). In particular, two sources of information were considered: (1) the number of Web pages annotated by a user or within a group; (2) the correspondence between the terms identifying the interests of a group and the tags that users attach to their annotations.

The fundamental assumption was then made that the focus of a group could be represented by a domain ontology, given as a graph of terms linked by semantic relations. Based on this, two classical measures associated with ontology graphs were considered: *Class Match Measure (****CMM****)* and *Degree Centrality (****DC****)*, matching tags present in annotations produced by users to terms in ontologies. While **CMM** simply compares the terms in the ontology with the tags employed by users in their annotations, **DC** takes into account the relevance of relations, searching for the maximal connected subgraph for the matched terms.

Experimental results showed that either of these measures succeeded, on the whole, in matching user interests and group topics, with **DC** being faster and producing more accurate results than **CMM** (Avola et al., 2014a). However, when results based on these measures were ranked, the quality of the rankings was found wanting when compared with manual inspection of the associated annotations—i.e., the groups (or users) that should have ranked highest were not those appearing in the first positions in the ranked list of recommendations. One inherent limitation is that these rankings could only be based on information associated with annotations and visited Web pages, since no relevant personal information and no activity besides annotation is recorded for MADCOW users, different from what occurs on e-commerce portals or in social networks, where a range of activities stem from visiting a page and are captured by the system. Although the experimental tests in Avola et al. (2014a) were mainly concerned with comparing the performances of **CMM** and **DC** when used in isolation, they provided some information about the ranking process itself, as shown in Table 1. The percentage of participants who selected either the first or the second recommended group was about 60% in the case of **CMM** and near 90% for **DC**. Similarly, when picking up users for inviting them to groups, the percentage of participants who selected either the first or the second recommended user was about 50% in the case of **CMM** and above 80% for **DC**.^2^

**Table 1:** 1st and 2nd selections for users and groups suggestions.

Degree centrality measure	Users suggestion percentage	Groups suggestion percentage
CMM	20%, 30%	45%, 15%
DC	66%, 16%	75%, 14%

This motivated us to call into question the ranking process as such, analyzing the limitations of those measures. Our findings confirmed that such limitations exist, showing that rankings based solely on semantic similarity lead to suboptimal recommendation quality.

The following two simple examples illustrate typical problems.

**Example 1**. Consider a situation where user U1 submitted one public annotation with seven different tags, and user U2 submitted five public annotations with just a single tag for all of them. Suppose that the annotations of both users are related to the same domain and are compared with terms for a given group G. Based on **CMM** or **DC** alone, U1 will rank higher than U2, although the latter is more active in the system and could be more useful as a member in group G.

**Example 2**. Consider a situation where two groups, G1 and G2, are both associated with a domain D. When matched with the interests of user U3, whatever they are, they will be equally ranked with respect to the domain, even though they might differ in the number of members, average activity of members, number and recency of annotations, all elements which might be relevant to decide whether to join a group.

To sum up, the original ranking mechanism did not take into account the following factors:

1. *User activity* in terms of:
  - Number of submitted annotations;
  - Number of joined groups.
2. *Group relevance* in terms of:
  - Number of group members;
  - Group membership growth rate.

Recent advances in decision-making under uncertainty further emphasize the necessity of taking into account more than just semantic similarity. In particular, fuzzy-based and multi-attribute decision-making methods have been successfully applied in many different domains. For instance, fuzzy extensions of the SERVQUAL model have been utilized to capture subjective impressions and ambiguity in user ratings in order to assess quality in primary healthcare settings (Santos et al., 2026). Similarly, by merging several competing criteria, fuzzy aggregation operators, such as Fermatean fuzzy Archimedean Heronian mean models, have been employed to enhance sustainable urban transport decision-making (Firat Kayiran, 2025). In the context of smart cities, multi-attribute group decision-making strategies have also been proposed to enhance public engagement, such as selecting digital voting tools for urban transport planning (Hussain et al., 2025). Furthermore, engineering disciplines have studied machine learning-assisted analytical methods, such as thermo-elastic analysis of composite structures (Kakati et al., 2024), to characterize complex systems under uncertainty.

While these techniques demonstrate the successful integration of fuzzy logic and multi-criteria reasoning, they are not immediately relevant to annotation-based recommender systems due to the requirement that recommendations be derived from limited and implicit user-generated input. In particular, they do not address the issue of ranking users and groups based on both semantic relevance and behavioral dynamics. This emphasizes the need for a unified framework that can incorporate semantic similarities with user activity and group characteristics, as proposed in this work.

This research examines how to improve recommendation quality by jointly modeling semantic, behavioral, and structural factors in annotation-based systems. Specifically, we address the following research questions:

- **RQ1** How can semantic similarity, user activity, and group-level characteristics be effectively integrated to improve user–group recommendation quality?
- **RQ2** How can a ranking mechanism be designed to better reflect real user engagement and group relevance beyond ontology-based matching?

This study provides clear answers to the above research questions. In response to **RQ1**, we propose a unified framework that integrates ontology-based semantic similarity with behavioral indicators (e.g., user activity) and group-level characteristics (e.g., group size and growth dynamics) using a fuzzy logic-based aggregation mechanism, enabling a more comprehensive modeling of user–group relevance. In response to **RQ2**, we design a fuzzy logic-based ranking mechanism that combines these heterogeneous factors under uncertainty, allowing the system to better capture real user engagement and group relevance beyond traditional ontology-based matching approaches. The effectiveness of these design choices is further supported by the experimental results presented in this work.

The proposed approach enables a more realistic and effective ranking of users and groups, improving the likelihood of meaningful participation and sustained engagement. In particular, we resort to fuzzy logic to provide suitable weights to the outcomes of different results.

Within a single decision-making model, the suggested ranking framework combines behavioral indicators like user activity and group characteristics with ontology-based semantic similarity. By intrinsically handling the ambiguity and imprecision contained in user-generated annotations through the use of fuzzy logic, the method allows a more accurate representation of relevance. By taking into account both semantic alignment and engagement-related characteristics, this integration improves suggestion quality and produces a more efficient ranking of individuals and groups.

Overall, the proposed method provides a flexible and extensible framework for improving recommendation quality in annotation-based collaborative environments, while also highlighting trade-offs between accuracy, interpretability, and computational efficiency.

The remainder of the paper proceeds as follow. After considering related work in Section 2, we describe the reasons for adopting a fuzzy logic framework in Section 3, and discuss its usage for refining rankings in Section 4, illustrating the matter with examples in Section 5. Section 6 describes the system architecture, while Section 7 presents a realistic scenario of usage. After reporting on experimental tests in Section 8, we conclude and discuss future work in Section 9.

## 2 Related work

With the diffusion of Internet-based protocols, collaborative sessions between people across remote sites are becoming increasingly frequent and are considered an appealing way to exchange ideas, thoughts, and experiences. Enriching resources at different sites with informative notes is a well-known practice in groupware environments. In these contexts, security and privacy were soon considered important issues for participants and were addressed in services for groups (Kumar and Varma, 2002).

In the context of smart cities, users and city stakeholders annotate data about services like transportation, environmental monitoring, and community planning, on digital platforms designed to facilitate citizen engagement, participatory governance, and shared urban data exploration (Bastos et al., 2022).

Online annotation tools enable collaborative activities via annotation threads. Few of these tools support groups as a method for creating focused discussions among participants sharing similar interests; on the contrary, most of them support only public annotations. Among those offering groups, little support is given for group management, thus hindering the transfer of annotations among groups, nor is the mutual relevance of users and groups measured, which we assume to be important for collaborative work.

Among these tools, Diigo Toolbar^3^ lets users create groups and invite other members, and provides some services for group archiving and dissemination. Annotate^4^ takes a snapshot of a document, Web page, or image, to produce a read-only copy, which can be annotated and shared with other users, depicting a kind of grouping. In Bounce,^5^ users produce and collaborate on notes for snapshots of Web pages.

Annotation and knowledge-sharing tools integrated into urban platforms have been the subject of recent research on smart city applications. These tools allow participants to annotate city data and interact with communities of practice that are focused on particular urban topics like energy consumption, mobility trends, or public services (Deligiannidou et al., 2016).

Ontologies as representatives of knowledge were used in several domains. Paralic and Kostial (2003) considered ontologies as representational schemes for domain knowledge in information retrieval and compared them with vector and latent semantic indexing models. Patel et al. (2007) proposed ontologies for the automation of common clinical tasks, e.g., selection of a patient cohort for clinical trials, considering the matching of patient records to trials as a problem of semantic retrieval.

Ontologies are used in the field of smart cities to semantically express various urban concepts and promote data source interoperability, enabling systems to semantically match groups or people with similar interests (Pliatsios et al., 2023).

Recent advances in smart city applications have relied on fuzzy logic and multi-criteria decision-making in addressing complex urban optimisation problems. For instance, AI-driven energy optimisation in smart homes has been studied using interval-valued Fermatean fuzzy Aczel–Alsina aggregation operators, enabling efficient handling of uncertainty in energy consumption patterns and decision parameters (Senapati et al., 2025). Similarly, in the domain of smart logistics, prioritization of AI-based material handling approaches in sustainable warehouses has been addressed using a *q*-rung orthopair fuzzy CoCoSo methodology with consensus-reaching mechanisms, supporting structured decision-making under multiple conflicting criteria (Saha et al., 2025).

These studies demonstrate the growing applicability of advanced fuzzy-based models in smart city environments, particularly for optimisation and resource management tasks. However, they rely on well-defined decision attributes and explicitly modeled criteria, which differ fundamentally from annotation-based systems where user preferences and interactions are implicitly derived from unstructured and sparse behavioral data. Therefore, while these approaches are effective for structured smart city optimisation problems, they are not directly applicable to recommender systems that require joint modeling of semantic similarity, user activity, and group dynamics.

Recent advances in decision-making for smart city environments further emphasize the importance of multi-criteria and fuzzy-based reasoning. For example, multi-attribute group decision-making methods have been proposed for participatory urban decision processes, such as the selection of digital voting tools aimed at improving citizen engagement in urban transport systems (Hussain et al., 2025). In addition, fuzzy aggregation approaches, including Fermatean fuzzy Archimedean Heronian mean models, have been applied to address sustainable urban transport planning problems involving multiple conflicting criteria and uncertainty (Kakati et al., 2024). These studies highlight the relevance of structured decision models in urban environments, but they rely on explicitly defined attributes and decision matrices, which differ from the implicit and unstructured nature of annotation-based recommender systems.

Several works deal with defining criteria to measure the relevance of an ontology to a collection of terms. In Cordì et al. (2005), similarity measures are defined for sets of concepts belonging to a common ontology, namely, similarity between a single concept and a set of concepts, as well as between two sets of concepts, establishing some criteria that should be met.

Prioritization and personalisation of information have increased the demand for recommendation systems, helping to make sense of the tremendous amount of information available on the net. Recommendation systems generate lists of items ranked with respect to some criterion (Isinkaye et al., 2015) as a decision-making strategy for systems acting on complex information environments (Rashid et al., 2002). In Isinkaye et al. (2015), the characteristics and potentials of different prediction techniques in recommendation systems are explored at length to serve as a compass for research and practice in the field. They describe the typical behavior of a recommender system as a sequence of phases, starting from information collection and explicit/implicit/hybrid feedback phases, followed by the use of filtering techniques such as content-based and collaborative filtering, and ending with some evaluation metrics for recommendation algorithms.

Beyond recommender systems, fuzzy logic has also been widely applied in domains involving subjective evaluation and uncertainty. For instance, fuzzy extensions of the SERVQUAL model have been used to assess service quality in primary healthcare systems by incorporating linguistic uncertainty and user perception variability (Mekuria, 2023). While these approaches demonstrate the effectiveness of fuzzy reasoning in capturing subjective judgments, they assume structured evaluation inputs and explicit feedback, which are not available in annotation-based environments where user preferences must be inferred indirectly from behavioral traces and semantic annotations.

Recommender systems are increasingly used in smart city research to enable participatory decision-making, personalize citizen services, and direct users to pertinent city information or urban communities.

The move from single-item recommendation-based systems to group ones is discussed in Felfernig et al. (2018), where several group recommendation algorithms are presented, categorized under various paradigms, such as: *Preference Aggregation Strategy, Preferences Known Beforehand*, etc.

Of interest here are: *Collaborative Filtering for Groups*, based on measuring the similarity of items using a nearest neighbors technique in which the characteristics of items are studied, which are grouped based on the similarity between their characteristics; *Content-Based Filtering for Groups*, in which new items are recommended if their categories are similar to those preferred by the current users; and *Hybrid Recommendation for Groups*, aimed at compensating specific limitations of one recommendation approach with the strengths of another one.

The use of fuzzy logic as a methodology for constructing recommender systems was described in Yager (2003). However, such systems were not studied from a collaborative filtering point of view, but recommendations were based only on the preferences of the single individual for whom they were provided, i.e., without considering the preferences of collaborators. The authors called these preferences *reclusive* methods, seeing them as complementary to *collaborative* methods.

Conversely, the use of fuzzy logic to create ontologies is an established technique. In Calegari and Ciucci (2006), fuzzy ontologies were defined by directly integrating fuzzy logic in classical computational ontologies to deal with uncertainty reasoning problems and to consider the nuances of natural languages.

The work in Calegari and Sanchez (2007) shows how a fuzzy ontology-based approach can improve semantic document retrieval. After formally defining a “fuzzy knowledge base,” they discuss a special type of non-taxonomic fuzzy relationships, called “(semantic) correlations.” These correlations, first assigned by experts, are updated after querying or upon insertion of a document into a database.

A unique path can then be derived, connecting the entities involved in the query, which identifies the maximal semantic associations in the knowledge domain.

Recommender systems strongly depend on the quality of their ranking component. Several works discuss the embedding of fuzzy logic within ranking functionalities to take into account the uncertainty in the measure of the properties involved in ranking (Nowacka et al., 2008). The work in Laughlin et al. (2011) discusses a study on personalized add-on filters applied to Web search results to make those results more intuitive to users. Fuzzy set theory and fuzzy logic are used to construct such filters, extracting linguistic features as their universe of discourse. Three experimental filters were presented: *narrowing down results, product specification*, and *tutorial-level classification*. The work in Chkiwa et al. (2013) proposed a collaboration between Semantic Web and fuzzy logic, seen as interoperable axes in the information retrieval process, to handle uncertainty to cover more relevant items. Fuzzy rules were used to enhance the query background expression and the ranking process.

Work related to semantic user and community recommendation in smart city platforms is also emerging, focusing on how to connect users with relevant urban information or urban communities based on semantic and activity features (Gomes et al., 2012).

Few works deal with the usage of recommender systems within annotation processes are limited. Among those, we mention (Pliakos and Kotropoulos, 2015) on building an image annotation and tourism recommender system. They propose a complete image annotation and recommender system based on probabilistic latent semantic analysis (PLSA) and hypergraph ranking, exploiting the visual attributes of the images and the semantic information from image tags and geo-tags. In their work, semantic image annotation resorts to the PLSA, exploiting the textual information in image tags and complementing it by visual annotation based on visual image content classification. Tourist destinations, strongly related to a query image, are recommended by using hypergraph ranking enhanced by enforcing group sparsity constraints.

Complementary machine learning-assisted analytical methods have been explored in engineering domains to model complex systems under uncertainty, such as thermo-elastic analysis of fiber-reinforced composite structures (Firat Kayiran, 2025). Although these methods effectively combine data-driven modeling with physical system analysis, they operate in environments with well-defined quantitative data and cannot be directly transferred to annotation-based recommender systems, where data is sparse, implicit, and semantically driven. This highlights the need for approaches integrating semantic reasoning with behavioral and structural information in a unified framework.

Overall, existing approaches either focus on structured multi-criteria decision-making under explicit assumptions or on semantic recommendation without behavioral modeling, leaving an open gap in combining both aspects for annotation-driven collaborative systems.

In this work, we improve the user-groups recommendation component originally implemented within MADCOW (Avola et al., 2014a) by integrating the processes of ranking users (resp. groups) toward groups (resp. users) with the use of fuzzy reasoning techniques. This is achieved by re-ranking lists of users/groups according to additional properties associated with linguistic variables on which to perform fuzzy reasoning.

## 3 Linguistic modeling

To assess the usability of the mechanisms for group collaboration presented in Avola et al. (2017), we enrolled 152 students from 8 undergraduate classes of different disciplines into a supervised test extending over 15 days. Some of the participants were asked to create groups, while others were allowed to submit annotations after joining a group.

**Figure 1:**
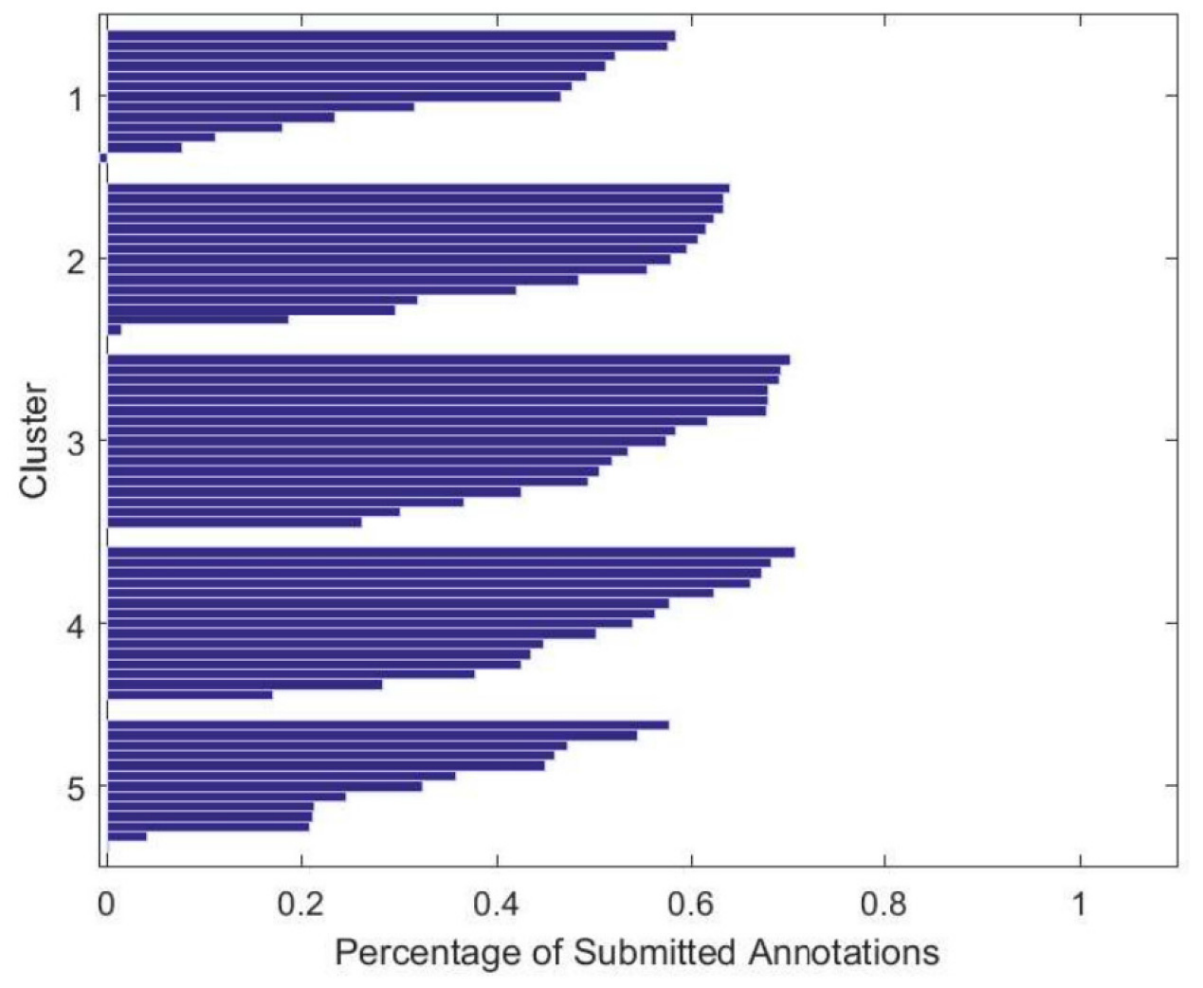
Clusters of submitted annotations generated by elbow and *k*-means algorithms.

Derived from the data collected in those tests, Table 2 classifies users according to the number of annotations they submitted, while Table 3 classifies them according to the number of groups they joined. The intervals displayed in the two tables are determined by Matlab implementations two algorithms. First, the Elbow method (Bholowalia and Kumar, 2014) (a heuristic technique based on inspection of plots of variance toward the number of clusters) was used to obtain the best number of clusters to detect in a collection. Then, the *k*-means algorithm (a technique of unsupervised clustering that classifies a collection of data objects into *k* disjoint clusters) was applied taking as value for *k* the one obtained by the Elbow method. As a sample of the generated clusters, Figure 1 depicts the 5 clusters related to the submitted annotations obtained according to the above procedure.

**Table 2:** Number of users for number of annotations submitted.

# Of annotations	# Of users
1–5	48
6–10	22
11–15	13
16–20	10
21 –25	5
>25	2

**Table 3:** Number of users for number of groups joined.

# Of groups	# Of users
1–3	64
4–6	20
7–9	16
10–12	8
13–15	1
>15	1

Apart from dealing with a limited number of users for a specific period, data in Tables 2, 3 can only provide some vague indication about user activity and group relevance.

Indeed, assigning an activity level to users or an overall relevance to groups based on these parameters is inherently affected by some imprecision, due to the necessity of mapping a precise numerical value, obtained by objective measures, to some judgment expressed in linguistic terms. This motivated us to use linguistic variables and fuzzy membership values to relate these measures to subjective judgments.

We also remark that the values in Tables 2, 3 portray the situation at the end of the period considered for the test and might be different if the test had lasted for a longer period or involved more users. Still, they indicate that user behaviors and group attractivity may significantly differ in normal contexts. These data have then been used to determine the pivots for fuzzy membership functions for the experiments.

### 3.1 Linguistic variables

Given a domain *D*, a *fuzzy subset* *A* of *D* (or fuzzy set *A* for short, when the domain is known) is characterized by a *fuzzy membership function* μ_*A*_:*D* → [0,1], where μ_*A*_(*d*) denotes the *degree of membership* in *A* of an element *d*∈*D*.

In fuzzy linguistic modeling, sets of *linguistic variables* are defined for some domain of discourse *D* and assume *linguistic values* to which fuzzy membership functions over that domain are associated. In particular, we defined a set of linguistic variables, one for each of the following *measures*, all considered to range over the domain ℕ of non-negative integers.^6^

1. *S*(*u*), number of annotations submitted by user *u*.
2. *M*(*u*), number of groups joined by user *u*.
3. *N*(*g*), number of members in group *g*.
4. *R*(*g*), group membership rate: average number of new memberships in *g* per day (throughout the test).^7^

We also defined three sets of linguistic values, together with a fuzzy membership function $\mu_{v}^{l}:D\to \left[0,1\right]$, for each measure (linguistic variable) *l* over *D* and each linguistic value *v*. In particular, we have:

- *Size* = {*Slightly, Little, Small, Big, VeryBig*} contains the linguistic values for the linguistic variables (1) *uAnn*, for the *S*(*u*) measure, (2) *uMem*, for *M*(*u*), (3) *gMem*, for *N*(*g*), and (4) *gRte*, for *R*(*g*).
- *Actv* = {*Inactive, SemiActive, Active, VeryActive*, and *Super*} contains the linguistic values for the linguistic variable *uJdg*, used to express the overall judgment *A*(*u*) on the user activity, as derived from a combination of the values for the variables *uAnn* and *uMem*.
- *Relv* = {*Poor, Fair, Good, VeryGood*, and *Excellent*} contains the linguistic values for the linguistic variable *gJdg*, used to express the overall judgment *C*(*g*) on the group relevance, as derived from a combination of the values for the variables *gMem* and *gRte*.

In particular, the determination of the linguistic values *uJdg* and *gJdg*, expressing the two judgements, results from a combination of the values for the independent measures relative to users and groups, respectively, as will be described in the following through the definition of sets of discrete rules.

### 3.2 Recommending users to groups

As discussed above, deciding whether a MADCOW user *u* should be recommended for a group *g* involves assessing the level of user activity based on measures *S*(*u*) and *M*(*u*).

#### 3.2.1 Fuzzy sets and membership functions

**Figure 2:**
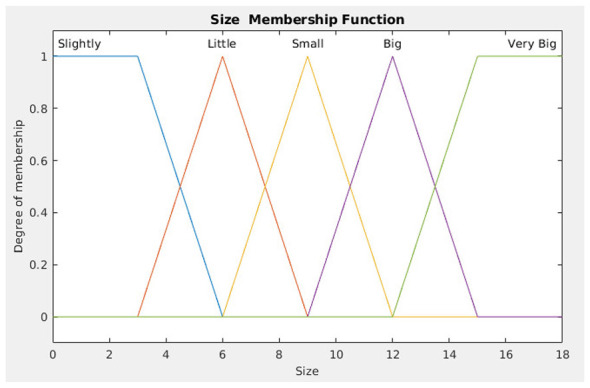
Fuzzy membership functions for the linguistic values in set size.

Based on the results in Tables 2, 3, we established pivots for linguistic values and used equally based triangular functions centered on the pivots, resulting in the fuzzy membership functions shown in Figure 2 and used for both of *uAnn* and *uMem*.

#### 3.2.2 Inference rules

The matrix *Mat*_*uJdg*_, shown in Table 4, supports the assessment of the value of the linguistic variable *uJdg*, where the membership levels for its possible linguistic values derive from the combination of the membership levels for the linguistic values of the variables *uAnn* (rows) and *uMem* (columns) referring to user activity. We will assign the values 1, 2, 3, 4, and 5 for the results: *Inactive, Active, Semi Active, Very Active*, and *Superb*, respectively. These values will be used in the defuzzificaion process in Equation 1. In particular, we adopt a *min*−*max* procedure, where the membership level for a linguistic value υ of *uJdg* is the maximum among all the values resulting from taking the minimum between the membership levels for the linguistic values, on rows and columns of *uJdg*, such that the corresponding cell presents the value υ. Formally, $\mu_{\upsilon }^{uJdg}=max\left\{\mu_{\rho }^{uAnn}\wedge \mu_{\gamma }^{uMem}|{Mat}_{uJdg}\left[\rho ,\gamma \right]=\upsilon \right\}$. Hence, for example, cell (*Little, Small*) in *Mat*_*uJdg*_ can be read as follows: If the number of annotations submitted by a user *u* is associated with the linguistic value *Little* with membership level $\mu_{Little}^{uAnn}\left(S\left(u\right)\right)=x$ and the number of groups *u* has joined is associated with *Small* with membership level $\mu_{Small}^{uMem}\left(u\right)=y$, then the value *x*∧*y* will be considered in the assessment of the membership level for the judgement of *u* being *SemiActive*.

**Table 4:** The matrix *Mat*_*uJdg*_ for judging user activity.

uAnn/uMem	Slightly	Little	Small	Big	VeryBig
Slightly	Inactive	Inactive	SemiActive	Active	VeryActive
Little	Inactive	SemiActive	SemiActive	Active	VeryActive
Small	Inactive	SemiActive	Active	Active	VeryActive
Big	SemiActive	Active	Active	VeryActive	VeryActive
VeryBig	SemiActive	Active	VeryActive	Superb	Superb

### 3.3 Recommending groups to users

For the process of suggesting groups to users, we are discussing two different factors that describe the relevance of a given group in the system:

1. Number of members inside the group.
2. Participation rate for the group.

Experimental results are synthesized in Tables 5, 6.

**Table 5:** Number of members inside groups.

# Of members	# Of groups
1–5	32
6–10	5
11–15	2
16–20	1
21–25	2
>25	8

**Table 6:** Membership rates for groups.

Daily average membership rate	# Of groups
1–3	9
4–6	7
7–9	1
10–12	2
13–15	1
>15	1

**Figure 3:**
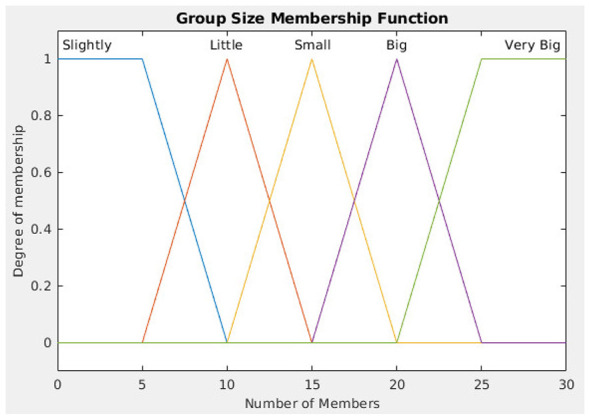
Membership functions for group size.

**Figure 4:**
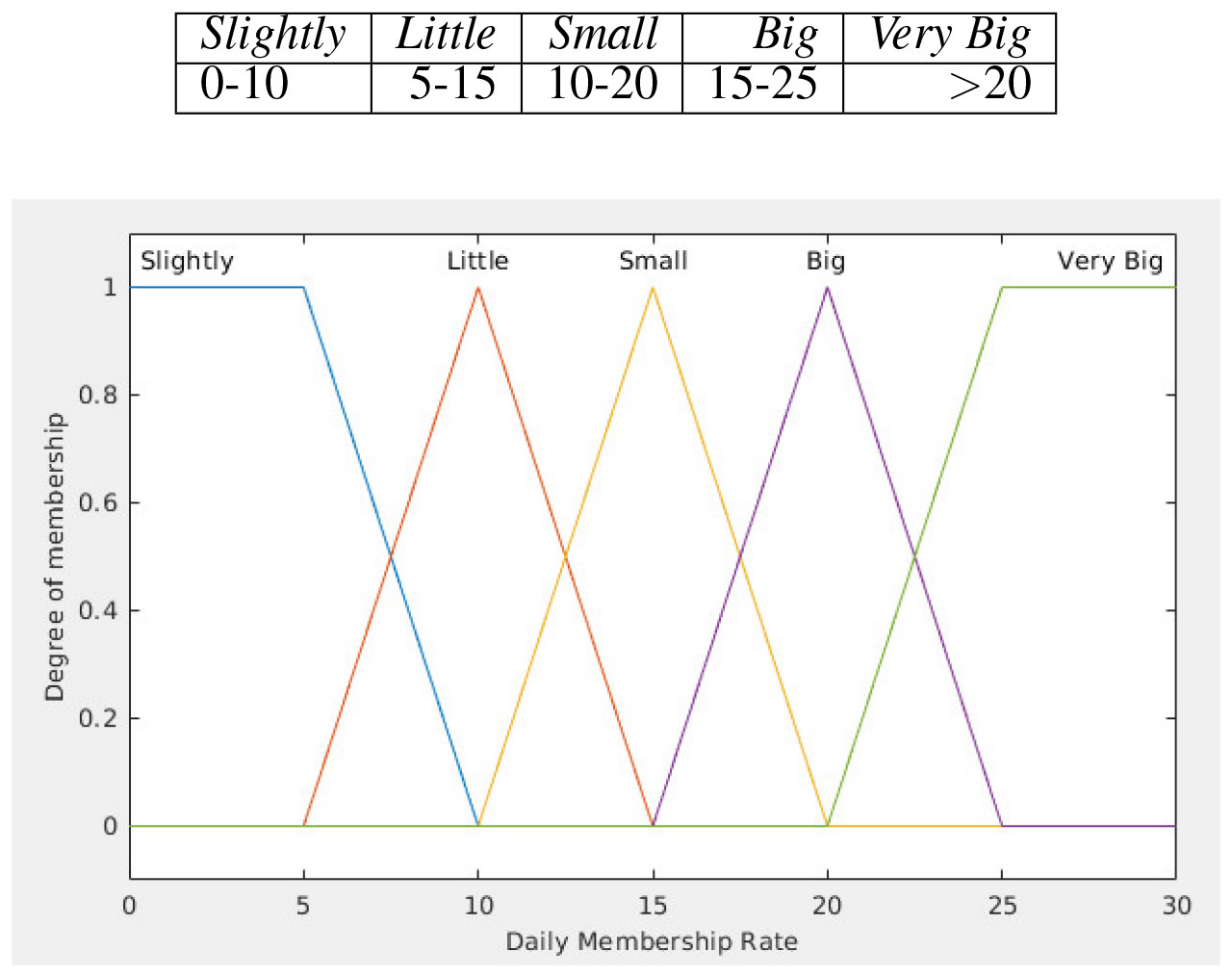
Membership functions for group growth rates.

From the classifications in Tables 5, 6 we associate linguistic labels with intervals as shown in Tables 7, 8; Figures 3, 4 provide a graphical representation of the respective fuzzy membership functions.

**Table 7:** Intervals for number of members in groups, *N*(*g*).

Slightly	Little	Small	Big	Very big
0–10	5–15	10–20	15–25	>20

**Table 8:** Intervals for growth rate in group size.

Slightly	Little	Small	Big	Very big
0–10	5–15	10–20	15–25	>20

#### 3.3.1 Inference rules

The matrix *Mat*_*gJdg*_, shown in Table 9, supports the assessment of the value of the linguistic variable *gJdg*, where the membership levels for its possible linguistic values derive from the combination of the membership levels for the linguistic values of the variables *gMem* (rows) and *gRte* (columns) referring to group importance. As in suggesting users to groups, we adopt a *min*−*max* procedure, where the membership level for a linguistic value υ of *gJdg* is the maximum among all the values resulting from taking the minimum between the membership levels for the linguistic values, on rows and columns of *gJdg*, such that the corresponding cell presents the value υ. Formally, $\mu_{\upsilon }^{gJdg}=max\left\{\mu_{\rho }^{gMem}\wedge \mu_{\gamma }^{gRte}|{Mat}_{gJdg}\left[\rho ,\gamma \right]=\upsilon \right\}$. Hence, for example, cell (*Little, Small*) in *Mat*_*gJdg*_ can be read as follows: If the number of members in a group *g* is associated with the linguistic value *Little* with membership level $\mu_{Little}^{gMem}\left(S\left(g\right)\right)=x$ and the membership rate of group *g* is associated with *Small* with membership level $\mu_{Small}^{gRte}\left(M\left(g\right)\right)=y$, then the value *x*∧*y* will be considered in the assessment of the membership level for the judgement of *g* being *Fair*.

**Table 9:** The matrix *Mat*_*gJdg*_ for judging group relevance.

gMem/gRte	Slightly	Little	Small	Big	VeryBig
Slightly	Poor	Poor	Fair	Good	VeryGood
Little	Poor	Fair	Fair	Good	VeryGood
Small	Poor	Fair	Good	Good	VeryGood
Big	Fair	Good	Good	VeryGood	VeryGood
VeryBig	Fair	Good	VeryGood	Excellent	Excellent

### 3.4 Logical operators on fuzzy sets

We combine the results of individual assessments through the **AND** fuzzy operator, given by the *min* function. Then, the *max* function is applied to the generated results.

### 3.5 Defuzzification process

To obtain the final crisp values for both user activity and/or group relevance, a defuzzification process is executed, using the *Center of gravity for singletons* formula, given by:


$$\begin{array}{c} CrispValue=\frac{\overset{n}{\underset{i=1}{\sum }}\mu_{i}\times u_{i}}{\overset{n}{\underset{i=1}{\sum }}\mu_{i}} \end{array}$$ (1)

where *u*_*i*_ is the corresponding value for the output variable of index *i*, μ_*i*_ is the membership function after accumulation for that variable, and *n* is the number of variables.

## 4 Refinement process

We discuss here how the use of linguistic variables to model the relevance of a group (resp. user) to a user (resp. group) can enhance the recommendation ranking process.

To this end, we adapt the formula presented in Opsahl et al. (2010) to the combination of the values of **DC** with those used to define the levels of activity (resp., relevance) for users (resp., groups).

The Degree Centrality Measure (**DC**) measures how important a concept is in an ontology in relation to its number of connections when the ontology is seen as a directed graph, according to Landherr et al. (2010); Ni et al. (2013); Zhuge and Zhang (2010).

Based on the idea of a maximal connected subgraph made up of ontology concepts or lexemes matched to group terms, we modify this definition to provide a measure of an ontology's relevance for a group^8^.

**Figure 5:**
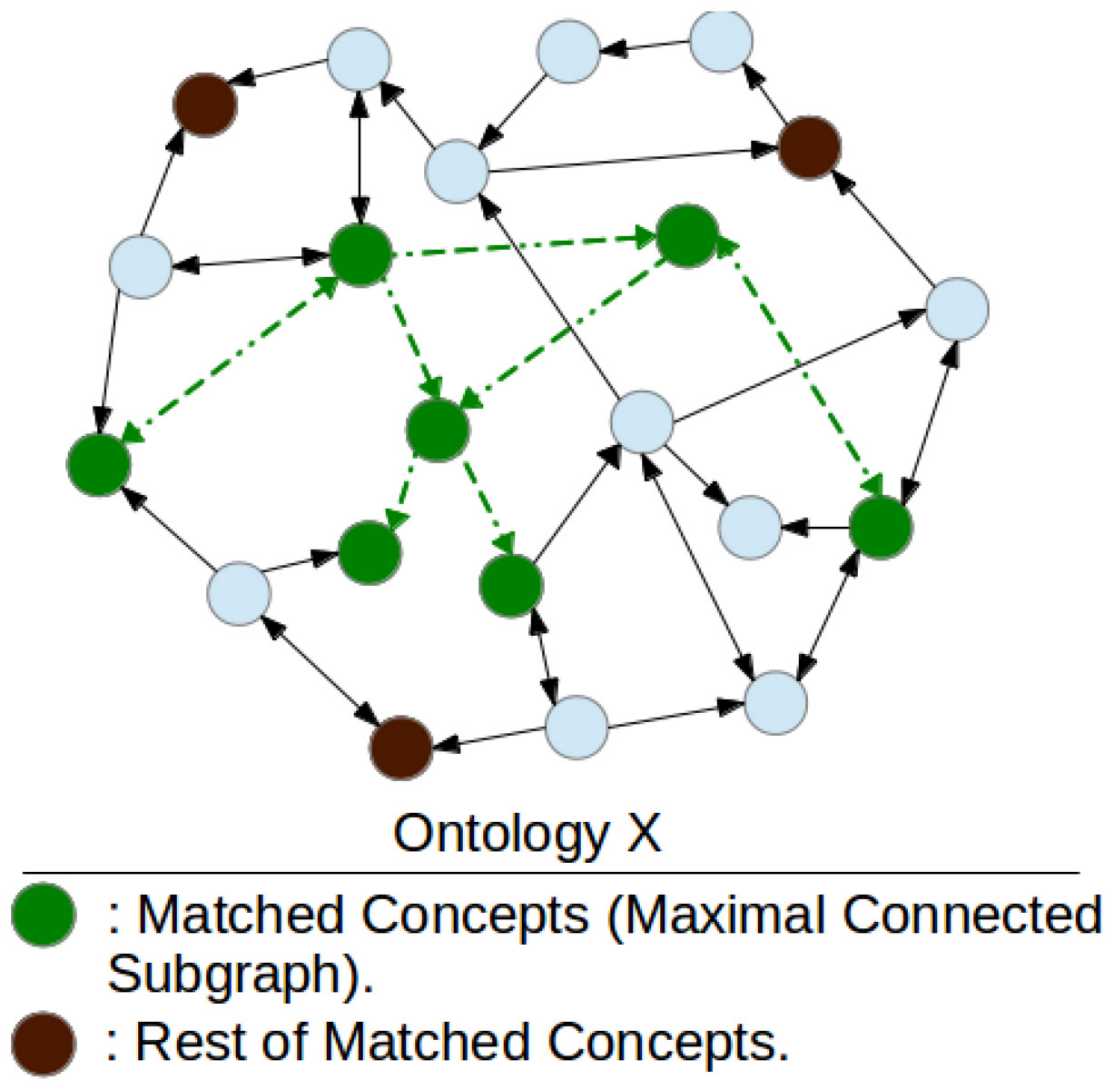
Ontology as a directed graph.

Figure 5 depicts an ontology as a directed graph, highlighting its maximal connected subgraph.

**Definition 1** (Matched concepts). With: *O* an ontology, *C*(*O*) the set of its concepts, *L*_*O*_(*c*) the set of lexemes for *c*∈*C*(*O*), and *T* a set of lexical items, the set *M*(*O, T*) of *matched concepts* is defined as:


$$\begin{array}{c} M\left(O,T\right)=\underset{t\in T}{⋃}\underset{c\in C\left(O\right)}{⋃}\underset{l\in L_{O}\left(c\right)}{⋃}H\left(c,l,t\right), \end{array}$$ (2)

where


H(c,l,t)={{c}if ∃ l∈LO(c) such that l=t0otherwise (3)

Given *M*(*O, T*), we build the graph *MG*(*O, T*) whose nodes are the elements of *M*(*O, T*). If the matching ideas are linked in *O*, then an edge exists between two nodes, *c*_1_ and *c*_2_. Let $MCSG_{v}^{i}\left(O,T\right)$ represent the collection of nodes for each maximal connected subgraph *MCSG*^*i*^(*O, T*) of *MG*(*O, T*).

**Definition 2** (Degree centrality of maximal connected subgraph). The *maximal degree centrality* for $MCSG_{v}^{i}\left(O,T\right)$ is defined as:

The ranking function is defined as shown in Equations 2–7.


$$\begin{array}{c} DC_{m}\left(O,T\right)=\underset{i}{\max}\left(\frac{\underset{c_{h}\in {MCSG}_{v}^{i}\left(O,T\right)}{\sum }MD_{i}-degree\left(c_{h}\right)}{\left(n_{i}-1\right)\left(n_{i}-2\right)}\right), \end{array}$$ (4)

where *n*_*i*_ is the number of concepts in the subgraph of index *i*, $MD_{i}=\max\left\{degree\left(c\right)|c\in MCS\overset{i}{\underset{n}{G}}\left(O,T\right)\right\}$, and


degree(c)=sub(c)+sup(c)+part_of(c)+sim(c),

is the number of concepts related to *c* via *Subclass, Superclass, Part-of* , or *Similarity* relationships.

In other words, with *MD*_*i*_ being the highest number of connections for a node in the maximal connected subgraph of index *i*, the value of *DC*_*m*_(*O, T*) is given by summing the differences in degree between *MD*_*i*_ and every other node in the same subgraph and taking the maximum over the set of such maximal connected subgraphs.

**Definition 3** (Outer degree centrality). Given *M*(*O, T*) and *MCSG*^*i*^(*O, T*), let


$$\begin{array}{c} Q^{i}\left(O,T\right)=M\left(O,T\right)\backslash MCSG_{n}^{i}\left(O,T\right). \end{array}$$ (5)

Then the *outer degree centrality* is defined as:


$$\begin{array}{c} DC_{o}\left(O,T\right)=\max\left(\underset{c\in Q^{i}\left(O,T\right)}{\sum }degree\left(c\right)\right). \end{array}$$ (6)

Finally, the overall degree centrality is defined as:


$$\begin{array}{c} DC\left(O,T\right)=DC_{m}\left(O,T\right)+DC_{o}\left(O,T\right). \end{array}$$ (7)

Taking the ranking of users as a basis, let *O*(*u, d*) denote the relevance of some domain *d* for a user *u*, based on ontological matching computed using **DC**, and let *A*(*u*) denote the fuzzy value defining the activity of *u* as computed by applying the set of fuzzy rules in Section 3, the values of both of *O*(*u, d*), *A*(*u*) being in the range [0..1]. Our problem here is how to combine both values in one formula, complying with the following qualitative requirements:

1. If the ontological matching alone is sufficiently discriminant between users for a given domain, this information will be considered more relevant than the one given by the level of activity of the user (*no subversion*).
2. If the ontological matching provides similar values for different users, then the user's activity level is used (*refining when needed*).

To formalize and operationalise these requirements, we define a function *rel*:**D**×**U** → [0..1], expressing the final level of relevance for a user concerning a domain, and we impose the following conditions on *rel*:

1. if *O*(*u*_*i*_, *d*)>>*O*(*u*_*j*_, *d*) then *rel*(*u*_*i*_, *d*)>*rel*(*u*_*j*_, *d*), i.e., the ontology-based ranking cannot be subverted.
2. if *O*(*u*_*i*_, *d*)≈*O*(*u*_*j*_, *d*)∧*A*(*u*_*i*_)>*A*(*u*_*j*_) for *u*_*i*_≠*u*_*j*_ with respect to all public annotations for both of *u*_*i*_, *u*_*j*_, then *rel*(*u*_*i*_, *d*)>*rel*(*u*_*j*_, *d*), i.e., the relative ranking depends on the activity levels.

We also need to consider how much the difference in ontological matches for the interests of users is a discriminant factor for a domain. Indeed, a small difference may have greater significance if the range of possible values for the given domain concerning existing users is narrow, while it will have less relevance if such a range is large. To this end, we compute an adjustment exponent *t*_*u*_ for each user *u*. In particular, let *max*_*O*_(*u*, **D**), *min*_*O*_(*u*, **D**) respectively be the maximal and minimal relevance ontological values (for *u*) of the domains in **D**. We define the value *t*_*u*_ as:


$$\begin{array}{c} t_{u}=\frac{max_{O}\left(u,D\right)-min_{O}\left(u,D\right)}{max_{O}\left(u,D\right)} \end{array}$$ (8)

so that it is also comprised in the interval [0..1]. The value of *t*_*u*_ quantifies the influence of *A*(*u*): if it is close to 1, then there is a wide range of values between *max*_*O*_(*u*, **D**) and *min*_*O*_(*u*, **D**), thus emphasizing the role of *O*(*u*, **D**) in the calculation of *f*(*u*, **D**). Conversely, if *t*_*u*_ is closer to 0, i.e., the values of *max*_*O*_(*u*, **D**) and *min*_*O*_(*u*, **D**) are nearly the same, then the relevance values do not affect the ranking process that much, so the emphasis goes to the activity in this case, i.e., the value of *A*(*u*) is emphasized.

Hence, for a user *u* and a domain *d* we define *f*(*u, d*) as:


$$\begin{array}{c} f\left(u,d\right)=A\left(u\right)\times {\left(\frac{O\left(u,d\right)}{A\left(u\right)}\right)}^{t_{u}} \end{array}$$ (9)

where *t*_*u*_ is a value in the range [0..1], which is determined dynamically for each user.

An analogous formula, using an adjustment exponent *t*_*g*_, could be applied to refine the rank of a group *g* in the lists generated for the matching between a user and a domain, using a function *g*:**U**×**G** → [0..1] to represent the relevance of a group to a user. The value *t*_*g*_ is determined at the time of list generation, where the maximum and minimum ontological relevance values are taken into account.

We extracted data from the BabelNet Ontology^9^ to construct a collection of 40 ontologies with four types of relationships: *Subclass, Superclass, Part-of* , and *Similarity*. Lexicographic and encyclopedic coverage of words is offered by BabelNet, a multilingual encyclopedic dictionary. Additionally, it has a semantic network formed with 'Babel synsets' that links concepts and named things in a vast network of semantic relations with over 23 million entries. Each concept is represented by a specific synset, composed of all the synonyms for that concepts in the languages covered by BabelNet.

In this work, the extracted knowledge is treated as a lightweight ontology, where each synset corresponds to a concept (i.e., a class or entity), and the relations between synsets correspond to semantic properties. This representation is conceptually aligned with the standard ontology model used in Semantic Web technologies.

We develop a Java-based ontology extraction program. Given a domain as input (used to define the ontology scope), the program identifies the most relevant BabelNet synset and recursively explores related synsets to incrementally construct the ontology. In the current implementation, to control complexity, the depth of the exploration process is limited to three levels.

To bridge the gap between our implementation and traditional ontology formalisms, the constructed ontology follows a triple-based representation model equivalent to the RDF data model. Specifically, each extracted relation is represented as a (subject, predicate, object) triple, where:

1. The subject corresponds to a source synset (concept),
2. The predicate corresponds to the semantic relation type (e.g., Subclass, Part-of),
3. The object corresponds to the target synset.

**Figure 6:**
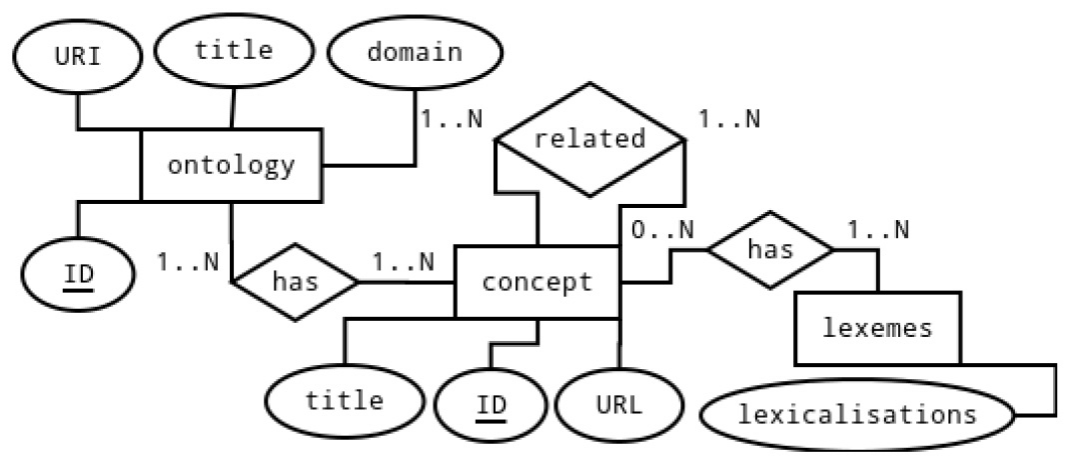
A fragment of the repository schema.


The proposed system adopts a hybrid approach, combining the formal semantics of ontology models with the scalability and performance of relational databases. For each visited synset, its identifier, URL, lexical representations, and relationships to other synsets, along with their types is stored in the database, physically realized using relational tables in MySQL for efficiency. This structure is fully compatible with standard semantic representations and can be directly mapped to RDF triples or serialized in formats such as Turtle. Indeed, the schema conceptually corresponds to a triple store representation, where relations between synsets encode the semantic structure of the ontology. This design allows efficient querying while preserving the semantic interpretation of the underlying knowledge graph. herefore,

A portion of the ontology repository's entity-relationship diagram is displayed in Figure 6. The Java implementation used to build the repository is shown in the following code sample. The structural features of the Ontology class are left out for simplicity.

Ontology ontology = new Ontology (name) ;
Concept cpt = create cpt (name , new BabConn ( ) ) ;
ontology . addConcept ( Cpt) ;
ontology . buildOnto ( Cpt . getLinkedConcepts ( ) ,
3 ) ;
saveInDataBase ( ontology ) ;
Concept create C p t ( String title , BabConn
babelNet ) {
ID = babelNet . getSynsetID ( title ) ;
URL = babelNet . getSynsetURL ( ID ) ;
lexemes = babelNet . getLexemes ( ID ) ;
linkedConcepts = babelNet . getLinked
Concepts ( ID ) ;
}
class Ontology {
. . .
public buildOnto ( L i s t <Concept > linked Cpts
, int level ) {
if ( level == 0) return ;
else {
List <Concepts > leaves = new
Array List ( ) ;
for ( Iteratorit = linked Cpts .
iterator ; it . hasNext ( ) ) {
Concept cpt = ( Concept )
( it . next ( ) ) ;
this . addConcept ( cpt ) ;
leaves . add ( cpt . getLinked
Concepts ( ) ) ;
}
this . buildOnto ( l e a v e s , −−l e v e l ) ;
}
}
}


The construction of Concept instances relies on a babelNet object to access BabelNet and retrieve all data related to a concept, as defined in the schema in Figure 6. After initializing the ontology with a root concept, it is expanded by recursively exploring related concepts from BabelNet.

The initial call to buildOnto uses the set of concepts linked to the root concept. Recursive calls continue exploring linked concepts up to a predefined depth (level 3 in this implementation). The function getLinkedConcepts retrieves concepts that have *Subclass, Superclass, Part-of* , or *Similarity* relationships with the current concept. Finally, saveInDataBase stores the constructed ontology in the database according to the relationships and properties defined in Figure 6.

In our paradigm, annotations are explicitly stored within the ontology structure rather than being treated as external metadata. Specifically, we introduce an Annotation entity with a semantic relationship to ontology concepts (i.e., synsets). Each annotation is associated with a concept through a relation similar to an object property (e.g., hasAnnotation), where the concept represents the domain knowledge element and the annotation records user-provided data.

Annotations also contain additional user and group links. A user is connected to annotations via a relation such as createdBy, and groups are established by collections of phrases extracted from annotations. The textual content of annotations (tags) is represented as descriptive attributes (data properties), which are subsequently used in the relevance computation process. This architecture ensures that annotations are fully integrated into the ontology-inspired structure while maintaining compatibility with popular semantic formats like RDF and OWL.

Ontologies are modeled as domains, groupings by sets of terms selected by their owners and users, and by sets of tags used to decorate annotations, building on this representation. Based on **DC**, we define three distinct relevance metrics.

1. Relevance of the group domain: By assessing *DC*, where *O* is the ontology associated with a domain and *T* is the set of words *Tms*(*g*) the owner designates as characterizing the group *g*, this measure helps the group owners discover the relevant domains.
2. Domain-Users Relevance: Group owners can find possible members with the use of this measure. In this case, *T* is the set *Tgs*_*p*_(*u*) of tags in the *public* annotations of a user *u*, and *O* is the ontology that describes the domain to which the group is related.
3. User-Domains Relevance: Users can find suitable groups based on their interests with the help of this measure. The collection of tags *Tgs*_*a*_(*u*) = *Tgs*_*p*_(*u*)∪*Tgs*_*v*_(*u*) in a user *u*'s *public* and *private* annotations matches the ideas in *O*.

In each of these scenarios, domains or users are then prioritized based on the accepted measure's importance.

The pseudocode that follows^10^ explains how we rank the relevance of ontologies from a given collection for a group *G* represented by its words for **DC**. We assume that ontologies is an array with members that store data on the various ontologies. Nested loops are used in the code to make it easier to grasp. Loops are actually accomplished using the appropriate SQL commands.


ln = ontologies . length ;
ranksDC = new array[ln] of int;
relevanceDC = new array[ln] of float;
for(i = 0; i < ln; i++)
 relevanceDC[i] = matchDC(G.terms,  ontologies[i]);
 ranksDC = computeRanking(relevanceDC); 
 float matchDC(gTerms[], ontology) { 
 matched = checkMatch(gTerms[], ontology); 
 connected = checkMaxConnected(matched,
  ontology); 
 DC = match(connected, ontology);
  rest = matched - connected;
  for(j = 0; j < rest.length(); j++)
   DC += degree(rest[j], ontology); 
 return DC;
 }  
float degree(concept, ontology) {
  return sub(concept, ontology) +  
 sup(concept, ontology) +  
 part_of(concept, ontology) +  
 sim(concept, ontology);
 }


## 5 Illustrative example

**Figure 7:**
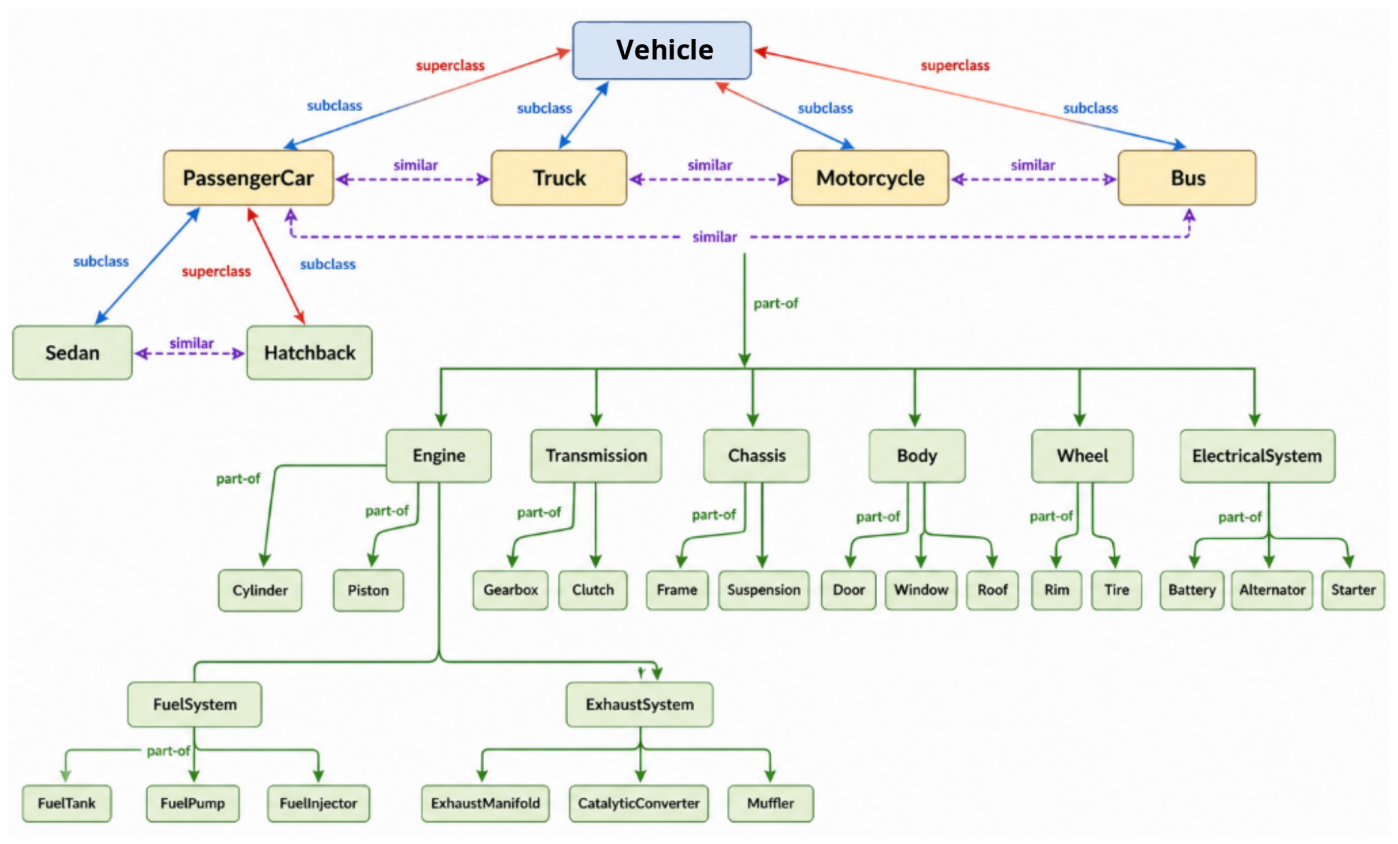
A fragment of vehicle ontology.

We now present an example showing the computational process leading to the production of a ranked list of users to a group owner. Suppose a MADCOW group *FeaturedVehicles* (*FV* for short), is mapped to some ontology *vehicle* (*O* for short), depicted (partially) in Figure 7. Now, suppose users *u*1, *u*2 have produced annotations from which two respective lists of tags have been extracted:

- **List 1**: *Body, Door, Window, Roof, Tire*,
- **List 2**: *FuelSystem, FuelTank, FuelPump, FuelInjector*.

Then, we hae $DC\left(FV,u1\right)=\frac{\left(5-1\right)+\left(5-1\right)+\left(5-1\right)}{\left(4-1\right)\left(4-2\right)}=2,DC\left(FV,u2\right)=\frac{\left(4-1\right)+\left(4-1\right)+\left(4-1\right)}{\left(4-1\right)\left(4-2\right)}=1.5$, so that *u*1 appears to be more relevant for *FV* than *u*2 ^11^.

Now, suppose that *u*1 has submitted 7 annotations and has joined 11 groups, while for *u*2 the corresponding values are 46 and 13. Using the functions in Figure 2, *u*1 is *small* with respect to number of submitted annotations and *big* for number of joined groups. On the contrary, *u*2 receives a judgement of *verybig* for both features. Based on Table 4, *u*1's *activity* is judged *Inactive*, while the judgement for *u*2 is *Superb*.

To compute the membership levels μ for both users, we refer again to the functions in Figure 2.

For *u*1 and submitted annotations:

-
$\mu \left(small\right)=\frac{7-6}{12-6}=0.166$

-
$\mu \left(little\right)=\frac{7-3}{9-3}=0.66$

For the number of joined groups:

-
$\mu \left(Big\right)=\frac{11-9}{15-9}=0.66$

-
$\mu \left(small\right)=\frac{11-6}{12-6}=0.83$

With reference to Table 4, and applying the *min*−*max* operations we have:

1. If *small* and *big* then *Active*, so 0.166∩0.66 = *min*(0.166, 0.66) = 0.166.
2. If *small* and *small* then *Active*, so 0.166∩0.83 = *min*(0.166, 0.83) = 0.166.
3. If *little* and *big* then *Active*, so 0.66∩0.66 = *min*(0.66, 0.66) = 0.66.
4. If *little* and *small* then *SemiActive*, so 0.66∩0.83 = *min*(0.66, 0.83) = 0.66.

As a result, we have: μ(*Active*) = *max*(0.166, 0.166, 0.66) = 0.66 and μ(*SemiActive*) = 0.66. Based on these values, we compute the relevance of *u*1 as $\frac{\left(2*0.66\right)+\left(3*0.66\right)}{0.66+0.66}=2.5$.

For *u*2 and submitted annotations: μ(*VeryBig*) = 1

For the number of joined groups:

- μ(*VeryBig*) = 1
-
$\mu \left(Big\right)=\frac{13-9}{15-9}=0.66$

Back again to Table 4 and applying the *min*−*max* operations we have:

1. If *VeryBig* and *VeryBig* then *Superb*, so 1.0∩1.0 = *min*(1.0, 1.0) = 1.0
2. If *VeryBig* and *Big* then *Superb*, so 1.0∩0.66 = *min*(1.0, 0.66) = 0.66

As a result, we have: μ(*Superb*) = *max*(1.0, 0.66) = 1.0. Based on these values, we compute the relevance of *u*2 as $\frac{\left(5*1\right)}{1.0}=5$.

Since the *DC* values are relatively close, we do not risk subversion and we can apply Equation 8, giving $t_{u}=\frac{2-1.5}{2}=0.25$. From Equation 9 we get $f\left(u1,FV\right)=2.5*{\left(\frac{2}{2.5}\right)}^{0.25}=2.36,f\left(u2,FV\right)=5*{\left(\frac{1.5}{5}\right)}^{0.25}=3.7$.

Hence, it results that *u*2 is more relevant than *u*1 to the group *FV*.

## 6 System architecture and behavior

**Figure 8:**
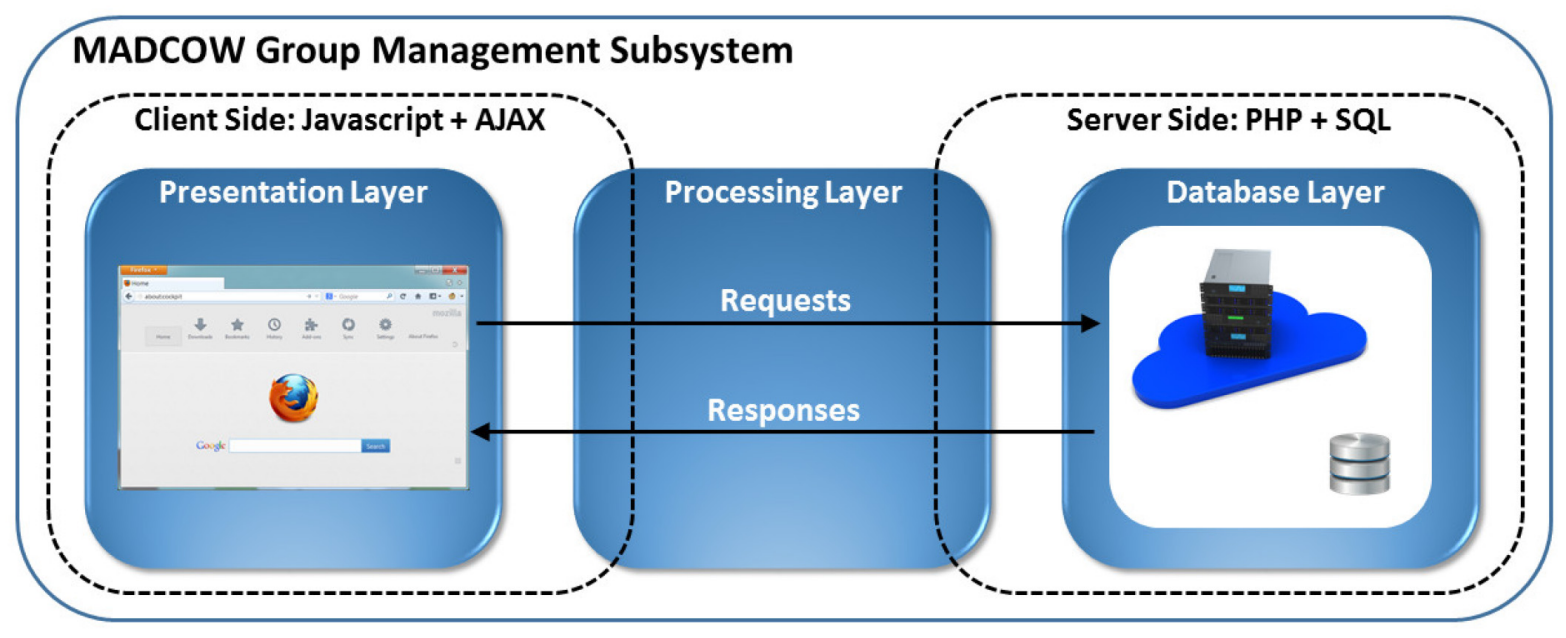
MADCOW architecture. The Presentation Layer deals with user interaction, generating requests to the server side. The Processing Layer, distributed between client and server, deals with information processing and the Database Layer manages information storage and responses to the client side.

As shown in Figure 8, **MADCOW** is a three-tiered client/server architecture Web-based system for annotation of HTML pages. The architecture follows the Model-View-Controller (MVC) pattern and a set of bookmarklets represents the core of the client side. Details about the architecture can be found in Avola et al. (2017), while in this paper we focus on the implementation of the recommendation process.

**Figure 9:**
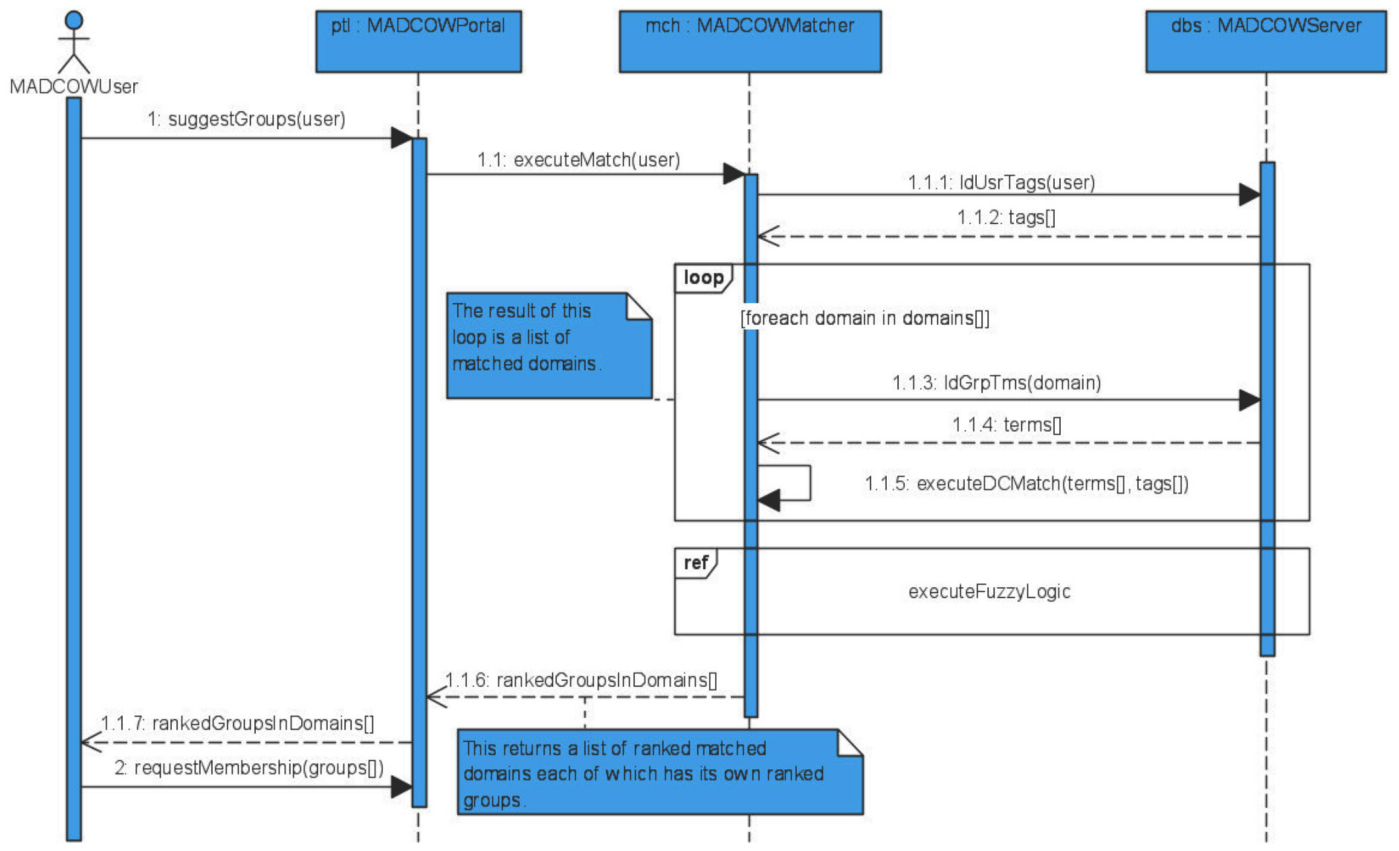
The sequence diagram for the process of suggesting groups to users.

Figure 9 depicts the sequence diagram specifying the process to suggest groups to users, using both **DC** and fuzzy logic. The interaction starts with a request for suggestions (suggestGroups(user)), to the MADOCWPortal object. This activates executeMatch(user) on MADCOWMatcher, which requests the MADCOWServer object, responsible for communication with the database, to retrieve the tags for the current user. MADCOWMatcher loops to obtain from MADCOWServer all the terms in each domain *D* from the database, and for each *D* matches its set of terms with the set of tags for the current user. If the match is above a certain threshold, *D* is added to matchedDomains[].

**Figure 10:**
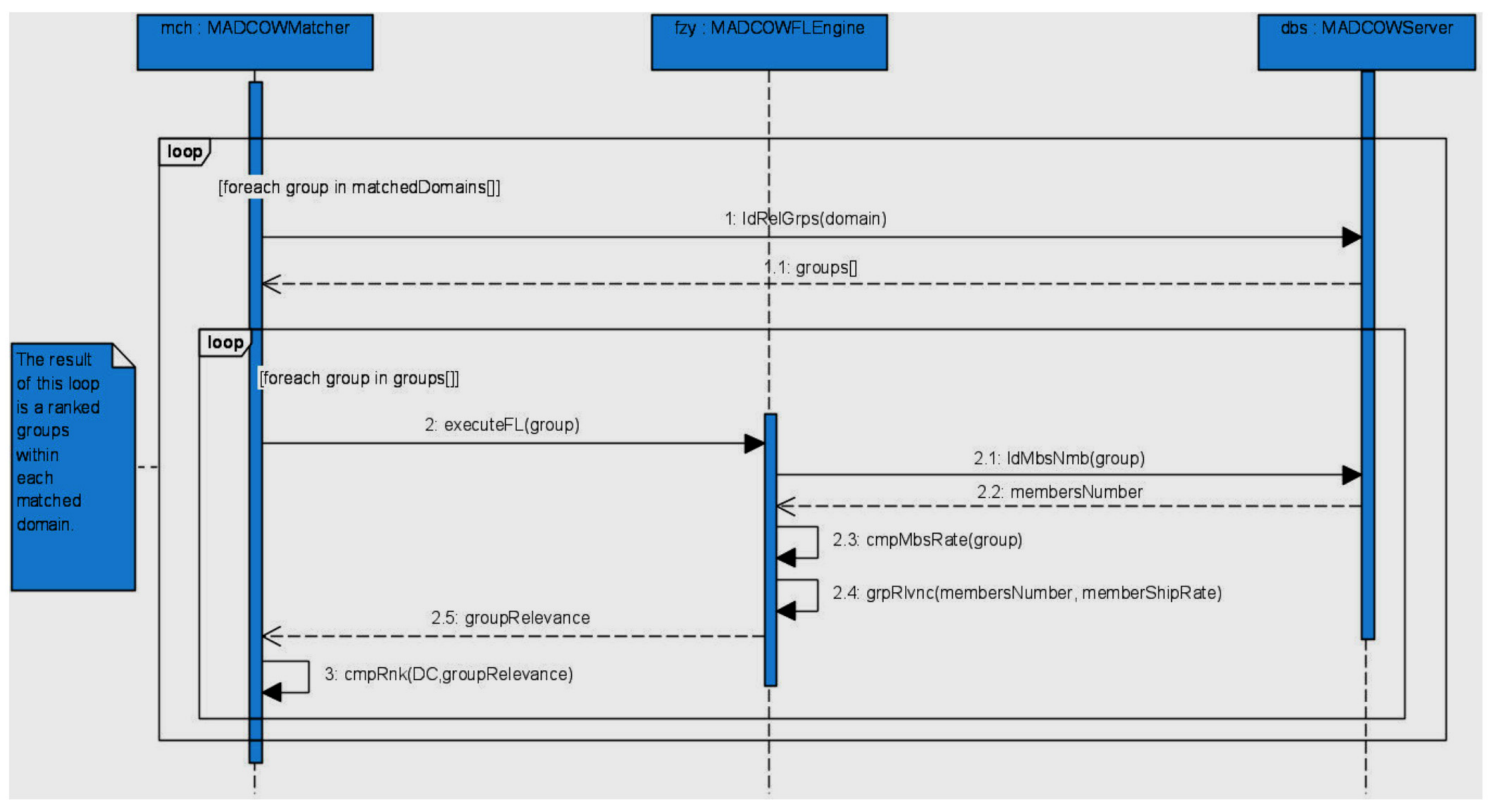
Sequence diagram for executeFL().

Figure 10 shows the iteration on each domain, where ldRelGrps(domain) is invoked on the server object by MADCOWMatcher to query the database, obtaining the list groups[]. For each group in groups, MADCOWMatcher then invokes executeFL(group) on MADCOWFLEngine, which queries the database for the size of the group (ldMbsNmb(group)) and calculates the group membership rate (cmpMbsRate(group)). Finally, group relevance is given by grpRlvnc(mbsNm, textttmbsRate). Then, MADCOWMatcher rank the groups within the current domain via cmpRnk(DC,groupRelevance).

In Figure 9, the list of groups thus produced is now used to generate a list of ranked domains, each of which has its own ranked groups, all saved in rnkdGrpsInDom to be returned to the object MADCOWPortal. The user can then select the groups to which to request the application, activating the function reqMbshp(groups).

The following pseudocode sketches the algorithm to compute the rank for each user in the process of suggesting users to a group owner using **DC** with the existence of fuzzy logic computation. In this pseudocode, the array ranks is used to store the final rank values of users; relevances stores the values of similarity between the given group and all system users (not members in that group); terms stores the group terms (loaded from the associated domain ontology); and the users array provides access to MADCOW users (not members of the group yet) for which relevance is being computed. The algorithm starts by loading the set of group terms into terms (using ldGrpTms()), while the array users is filled by executing ldSysUsrs(). Then, it iterates on all users to compute user similarity with each group. At each iteration, the system loads the tags of the current user into tags and, based on the content of terms and tags, computes the DC similarity (storing it in the *dc* variable).

Finally, the current user activity is assessed via cmpUsrAnns(user) and countGrpsMbshp(user), saving these values in the annCount and mbshpCount variables, respectively. Then, fuzzyValue stores the current user's activity level, as computed in computeFV(). This will be used together with DC to compute the relevance of the current user to the group by invoking emphasize()^12^ and stores its result in relevances. After the loop completes, the relevances array is analyzed in cmpRnks(), the result of which is finally stored into the ranks array and returned as the result.


int [ ] combinedWithFuzzySuggestUsers ( group ) {
 int[] ranks = {0};
  float[] relevances = {0}; 
 Term[] terms = ldGrpTms(group);
  User[] users = ldSysUsrs();  
foreach user in users {
   Tag[] tags = ldUsrTags(user);  
 dc = executeDC(terms,tags); 
  annCount = cmpUsrAnns(user);  
 mbshpCount = countGrpsMbshp(user);
   fuzzyValue = computeFV(annCount, 
 mbshpCount); 
  relevances[user] = emphasize(DC, 
 fuzzyValue); 
 } 
 ranks = cmpRnks(relevances); 
 return ranks;
 }


## 7 Working scenario

A smart city platform uses a framework to facilitate online conversations about city-related issues between residents, urban planners, and subject matter experts. By uploading information about local services, urban infrastructure, environmental monitoring, or community projects, users can engage with the system similarly to a social networking platform. The website also enables external stakeholders to provide their thoughts on posted urban data and discussion threads in order to use the expertise and knowledge of residents and specialists around the city. Discussions are carried out using **MADCOW** features, which allow for the organization of posted articles as discussion threads and the addition of notes.

Ms. Lina wishes to initiate cooperative conversations with other users who share her interest in Sustainable Mobility, a topic on which she has previously submitted multiple annotations. She thus requests that the **MADCOW** portal suggest organizations that concentrate on sustainable mobility. She uses **DC** alone to test the suggestions initially. She receives a list of groups in which various organizations working in the broad field of sustainable mobility are ranked equally.

Ms. Lina is perplexed by the results since she receives a number of groups that she knows are connected to her hobbies, but she is still unsure of which are most pertinent. She uses the **MADCOW** feature, which combines the **DC** measure with metrics of group relevance, membership growth rate, and user activity, to get a more targeted response on the annotations she supplied. The ranking that results enables her to find a few organizations that are concentrated on the subjects that are most important to her, such as *Bike Sharing Initiatives, Public Transport Optimisation*, and *Electric Mobility Community*.

## 8 Experimental tests

In this section, we report on some experimental tests to investigate the importance of introducing the implemented Fuzzy rules and functions to the recommendation part of **MADCOW**, comparing the quality of the received recommendations before and after introducing fuzzy logic to the ranking mechanisms.

### 8.1 Testing fuzzy sets (rank tuning)

In this test, participants are asked to compare the different ranks before and after applying the fuzzy functions. In both types of suggestions, two lists (different in ranking) are generated: one where ranking is based on the similarity measure alone and the other where ranking derives from combining the similarity measures and the properties discussed above.

#### 8.1.1 Testing preparation

Participants are divided into 3 sets: group owners, group members, and ordinary users (not enrolled in any group). Owners created a set of groups related to different computer science subjects and associated the relevant ontologies with them. Users in the second set are directly invited by owners to groups and begin submitting annotations to these groups. Users in the third set submit public and private annotations, together with informative tags.

#### 8.1.2 Testing process

After the creation of groups and direct inclusion of some users into them, users start submitting annotations (of the group, public, and private types) and group owners start asking the system to suggest members for their groups. Participants are asked to assess the quality of the enhanced process by recording the difference in ranks generated before and after applying the fuzzy functions compared with each similarity measure. Also, the same type of test can be executed for the process of suggesting groups to users. During the tests and after accomplishing each part of the tests, participants are asked to answer questions about their preference for the resulting rankings.

#### 8.1.3 Participants

Students of the course **“Human-Computer Interaction”** at An-Najah National University were selected to be participants in the experimental tests. They were given illustrative crash courses to be familiar with the usage of the system and with the need for the tests to have accurate results. They were introduced to the **MADCOW** system, trained to create groups and to invite users to them, and received a cursory introduction to similarity measures and their importance and usage in the recommendation process. They received complete information about the aim of the tests and the idea behind combining the similarity measures and introducing the fuzzy logic to the ranking of suggested groups and users.

Twenty three students were involved in the process and the illustrative lectures were conducted in the HCI Computer Lab of the Department of COmputer Science of An-Najah N. University^13^ in Palestine. After the initial presentation of the test activity, they were requested to execute the tests from their computers during the following two months. At the end of the test, participants were asked to fill out online questionnaires.

#### 8.1.4 Gathered data

At the end of the testing period, 45 groups had been created and associated with 14 different ontologies and 165 annotations had been submitted (45 public ones, and the rest submitted to groups).

Table 10 shows the average times (in seconds) for all matching operations conducted during the test. The values for average times appearing in the table are related to the number of textual matches occurring in the matching processes. In the case of suggestion of groups, the average number of provided terms is 5. In the case of suggestion of users, the average number of matches between terms and tags is: **DC** = 10. In the case of suggestion of groups: **DC** = 17.^14^ The reason that the number of matches for suggesting groups is bigger (in general) than for suggesting users is due to the involvement of both public and private annotations in the matching process for the first case, while in the case of user suggestions, only public ones are involved. The reader may notice that the fuzzy value associated with the suggestion of groups is omitted in the table, as fuzzy rules are not applicable in this operation.

**Table 10:** Average times calculated for all matching operations.

Operation	DC	
	Alone	Fuzzy
**Group Ass.**	1.151	–
**Sugg. users**	1.41	1.72
**Sugg. groups**	0.63	0.91

Table 11 represents the improvement in user selections compared with those in Table 1 in Section 1 with respect to **DC**. These data show that a higher percentage of selections were made on the first and second items from the generated lists when the fuzzy rules are involved, reflecting an improvement of the recommendation part of **MADCOW**.

**Table 11:** 1st and 2nd selections (after combination and fuzzy logic).

Combined measure	Users suggestion percentage	Group suggestion percentage
**DC & fuzzy**	75%, 35%	85%, 45%

#### 8.1.5 Tests

The testing session was composed of 2 tests:

1. Suggesting users (to invite) to group owners.
2. Suggesting groups to users.

Each test was conducted in a separate sub-session to ensure that participants executed the tests correctly. In the following, we illustrate in depth the execution of each test, together with the types of data generated and parts of the system involved.

### 8.2 Test 1: Suggesting users to groups' owners

The goal of the first test was to check if using a combination of **DC** and fuzzy logic did enhance the ranking of suggested users to group owners. Participants were divided into 3 disjoint sets (of size 8, 9, and 6, respectively). We asked participants of the first set to create some **MADCOW** groups (8 groups created) and to link them to appropriate ontologies (domains). After that, they could invite participants of the second set to join these groups (32 invitations were sent), and all invitations were accepted. The participants in the second set were then asked to start using **MADCOW** by annotating resources in 20 specially prepared Websites related to some extent with the subjects of the created groups. These participants then submitted the produced annotations to groups of their choice among those they were members of.

At the same time, the participants of the third set were asked to create public annotations on different websites and to attach some set of expressive tags to each annotation. The annotation process for participants in both the second and the third set lasted for two weeks (132 annotations were submitted).

After that, we asked group owners to use the **MADCOW** recommendation engine to look for potential, informing them that we needed to measure the enhancement of ranking after the introduction of fuzzy logic. For an easier comparison of the results, we programmed **MADCOW** to display the resulting list of suggested users onto 2 adjacent panels: results of **DC** alone and results of **DC** plus fuzzy logic.

To conclude, we asked group owners the question “Which is better: **DC** alone or **DC** with fuzzy logic?” Table 12 presents the percentages of answers, showing a preference for the introduction of fuzzy logic.

**Table 12:** Preferences of group owners.

Measure	Measure Alone	With fuzzy logic	No difference
**DC**	15%	74%	11%

### 8.3 Test 2: Suggesting groups to users

The second test involved only the participants who appeared in the third set for Test 1. They were asked to use **MADCOW** to obtain suggestions of suitable groups to join, based on the annotations they published. They were asked to use **DC** together with fuzzy logic in the suggestion process.

Hence, they were asked to evaluate the generated results in terms of the enhancement in ranking of domains and ranking of groups within each domain (a result domain could contain more than one group, since the comparison takes place between users tags and domain terms). At the end of the test, they were asked to answer the same question “Which is better: **DC** alone or **DC** with fuzzy logic?,” this time taking to consideration the suggestions they had received on which groups to join. Table 13 presents the percentages of answers, again showing a preference for the introduction of fuzzy logic.

**Table 13:** Preferences of annotators.

Measure	Alone	With fuzzy logic	No difference
**DC**	14%	76%	10%

## 9 Conclusions and future work

In this article, we addressed the problem of user–group matching in annotation-based recommender systems within smart city environments, leveraging the MADCOW framework.

The study is motivated by the need to improve recommendation quality in collaborative annotation systems, where traditional semantic matching approaches fail to capture the dynamic nature of user participation and group evolution. This highlights the importance of considering both content-based and behavioral factors in recommendation design.

Indeed, our experimental results showed that traditional ontology-based approaches relying on semantic similarity measures such as CMM and DC are insufficient to capture the full complexity of user and group behavior.

To overcome their limitations, we proposed a hybrid recommendation model that integrates semantic similarity with behavioral and structural indicators, including user activity (number of annotations and group memberships) and group relevance (group size and membership growth rate). In our model, these factors are combined using a fuzzy logic-based framework to support more flexible and realistic ranking decisions.

Experimental evaluation demonstrates that the proposed approach consistently improves recommendation quality for both *user-to-group* and *group-to-user* matching scenarios, outperforming traditional semantic-only methods in the majority of evaluated cases.

The results confirm that relying solely on semantic similarity is insufficient in dynamic collaborative environments. Instead, effective recommendation requires the integration of semantic, behavioral, and structural dimensions, which together provide a more accurate representation of user engagement and group evolution.

Despite its advantages, the proposed approach is affected by limitations related to: annotation sparsity and dependence on structured ontologies, which may reduce effectiveness in poorly populated or heterogeneous datasets; dependence on availability and quality of annotation data; need for well-defined ontologies; and increased computational complexity due to multi-factor fuzzy inference.

Future work will focus on addressing these limitations by exploring data-driven and adaptive enhancement strategies. In particular, hybrid recommendation models that combine annotation-based signals with additional contextual information (such as temporal activity patterns or implicit interaction logs) could mitigate sparsity effects. In addition, embedding-based representations and representation learning techniques may help capture latent relationships between users and groups beyond explicit annotations. Moreover, transfer learning approaches could be investigated to leverage knowledge from related domains, improving robustness in sparse environments. Finally, adaptive fuzzy membership learning strategies based on data distributions (rather than fixed thresholds or percentile heuristics) may further enhance scalability and real-world applicability.

Overall, this work contributes a unified fuzzy logic-based recommendation framework that combines semantic, behavioral, and structural information, leading to improved ranking performance and a more realistic modeling of collaborative user–group interactions in smart city environments.

## Data Availability

The original contributions presented in the study are included in the article/supplementary material, further inquiries can be directed to the corresponding author.

## Footnotes

1 **M**ultimedia **A**nnotation of **D**igital **C**ontent **O**ver the **W**eb.

2 To enhance readability and ensure clarity, we provide a complete list of the abbreviations used in the paper together with their full forms.

3
http://www.diigo.com/

4
http://a.nnotate.com/

5
http://www.bounceapp.com/

6 To enhance readability and ensure consistency, a comprehensive list of abbreviations is provided.

7 In this case we approximate the average to the closest integer number.

8 In case of multiple maximal subgraphs, we select the one with the highest **DC** value.

9
http://babelnet.org

10 Due to limitations on paper size, several functions were not coded.

11 *CMM* is not considered in this example because it is merged with *DC* in a previous work.

12 The implementation of this function includes the computation of *t*.

13
http://www.najah.edu

14 Of course, more matched terms means more consumed matching time.
